# The complete design in the composite face paradigm: role of response bias, target certainty, and feedback

**DOI:** 10.3389/fnhum.2014.00885

**Published:** 2014-10-31

**Authors:** Günter Meinhardt, Bozana Meinhardt-Injac, Malte Persike

**Affiliations:** Department of Psychology, Johannes Gutenberg University MainzMainz, Germany

**Keywords:** feature integration, composite effect, congruency effect, response bias, selective attention

## Abstract

Some years ago an improved design (the “complete design”) was proposed to assess the composite face effect in terms of a congruency effect, defined as the performance difference for congruent and incongruent target to no-target relationships (Cheung et al., 2008). In a recent paper Rossion (2013) questioned whether the congruency effect was a valid hallmark of perceptual integration, because it may contain confounds with face-unspecific interference effects. Here we argue that the complete design is well-balanced and allows one to separate face-specific from face-unspecific effects. We used the complete design for a same/different composite stimulus matching task with face and non-face objects (watches). Subjects performed the task with and without trial-by-trial feedback, and with low and high certainty about the target half. Results showed large congruency effects for faces, particularly when subjects were informed late in the trial about which face halves had to be matched. Analysis of response bias revealed that subjects preferred the “different” response in incongruent trials, which is expected when upper and lower face halves are integrated perceptually at the encoding stage. The results pattern was observed in the absence of feedback, while providing feedback generally attenuated the congruency effect, and led to an avoidance of response bias. For watches no or marginal congruency effects and a moderate global “same” bias were observed. We conclude that the congruency effect, when complemented by an evaluation of response bias, is a valid hallmark of feature integration that allows one to separate faces from non-face objects.

## 1. Introduction

A common observation in face perception or recognition experiments is that observers have difficulty judging face parts independently. In various studies, Tanaka and colleagues found that facial context strongly modulates recognition of face parts; for houses, researchers have observed less contextual influence (Tanaka and Farah, 1993; Tanaka and Sengco, 1997). The strong interdependence of parts in part-to-whole recognition and matching tasks led to the conclusion that faces are “special” compared to other object categories in that face processing involves relatively little part-based decomposition (Young et al., 1987; Tanaka and Farah, 1993; Farah et al., 1998). The stronger integration of parts for faces compared to non-face objects was substantiated in subsequent studies using classic hallmarks of feature integration (Gauthier et al., 1998; Yovel and Kanwisher, 2004; Kanwisher and Yovel, 2006; Robbins and McKone, 2007; Macchi Cassia et al., 2009; Taubert, 2009; Meinhardt-Injac, 2013).

Integrative processing of object parts may also arise and strengthen as a function of expertise, even with novel and artificial objects (Gauthier and Tarr, 1997). Testing selective attention to objects parts, Gauthier et al. (2003) found evidence that car experts had problems ignoring irrelevant car features. Further, the researchers found that the N170, a face-selective ERP component (Bentin et al., 1996; Itier and Taylor, 2004; Rousselet et al., 2004, 2008; Jacques and Rossion, 2009), was jointly modulated by cars and faces among car experts, which indicates that integrated encoding of object features may have a common sensory basis in objects of expertise. Later measurements failed to confirm similar results in measures of feature integration for faces and non-face objects of expertise, which led to criticism of the expertise hypothesis (Robbins and McKone, 2007). Albeit the dispute about the role of expertise there is consensus that faces and non-face objects differ in their degree of part integration when high degrees of familiarity, expertise or training are not involved (Gauthier et al., 2003; McKone et al., 2006; Rossion, 2013).

### 1.1. The composite face paradigm

**Figure 1:**
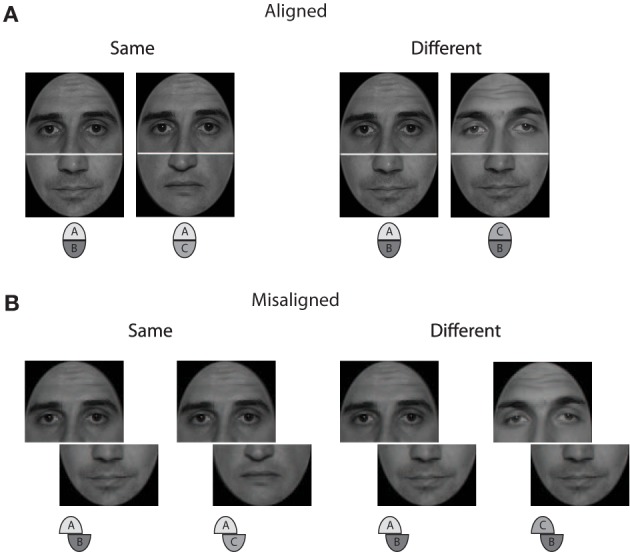
**Stimulus example for upper face half matching in aligned (A) and misaligned (B) arrangements**. The left composite face pair shows same upper halves combined with different lower halves, the right one shows different upper halves combined with same lower halves. Note that the example corresponds to a “incongruent” trial (see Figure 2) in the complete design.

A frequently used behavioral approach to measuring the degree of integration among face parts is the composite face paradigm (Young et al., 1987). In this paradigm, face composites are formed by combining a lower and upper half, both stemming from different persons. In the experiment, two such composite faces are shown and observers have to match either the upper or lower halves. Figure 1A illustrates matching the upper halves of two composite faces. When two upper halves are same with different lower halves (see “same” example in Figure 1), the upper halves look different. Because the two whole faces are indeed different, the failure to selectively attend to just one half may be because of perceptual integration among both halves (Rossion and Boremanse, 2008). Misaligning the halves hampers integration, and each one can be attended selectively (see Figure 1B).

**Figure 2:**
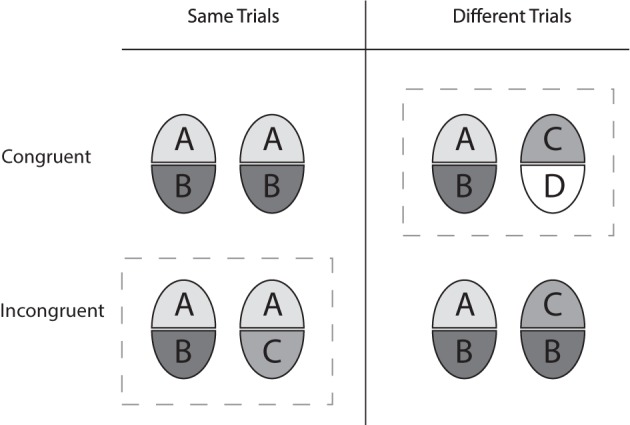
**Overview of the *complete design* and the *partial design* (elements within gray dashed boxes), according to Cheung et al. (2008)**.

In several studies the composite face paradigm was used in the following variety (Goffaux and Rossion, 2006; Rossion and Boremanse, 2008; Jacques and Rossion, 2009). In “same” trials the upper face halves were same while the lower ones were different. In “different” trials upper and lower halves were both different (see dashed gray boxes in Figure 2). Perceptual integration was concluded from the performance difference obtained for aligned and misaligned arrangements. The results of these experiments showed that strong modulatory effects of alignment existed for the “same,” but not for “different” trials. Therefore, the authors confined their analyses to the hit-rate (i.e., the rate of correctly indicating same face halves).

The particular way of defining same and different trials and the use of only the hit rate led to the criticism that non-perceptual strategies may have affected the results (Cheung et al., 2008). First, Cheung and colleagues argued that the frequency of same and different unattended face halves should be balanced to avoid induction of bias toward the “different” response category. As shown in Figure 2 (see gray boxes), the design used by Rossion and colleagues [called the “partial design” (PD) by Cheung and colleagues] includes more different halves than same halves, which might bias an observer's response strategy toward “different” responses. Second, they argued that, generally, any measure of feature integration should not be affected by an observer's response strategies. As with the theory of signal detection, they claimed that a bias-free measure of performance should be used. Such a measure can only derive from the performance achieved for both response categories (MacMillan and Creelman, 2005, p. 6).

To construct a design with an equal number of same and different halves they proposed to compose same- and different-trials in *congruent* (see 1st row in Figure 2) and *incongruent* (see 2nd row in Figure 2) variants, and referred to this partitioning as the “complete design,” CD. To use a bias-free measure, they proposed using *d*′, which is calculated from the relative frequency data of both response categories. Further, to measure how face halves interact the authors suggested using the performance difference achieved with congruent and incongruent trials, the *congruency effect* (CE). The authors pointed out that comparison of aligned and misaligned conditions is possible with the CD, but it is not necessary (Cheung et al., 2008, p. 1328), because the CE included the effect of interest with all aligned stimuli.

While performance in the congruent trials is widely unaffected by the global or local focus on the face stimuli, performance in incongruent trials can only be good if the observer can attend to only the target half and ignore the non-target half, as the non-target halves vary orthogonally to the target halves and are the same when the target haves are different and vice versa. An observer who is unable to selectively attend to the face halves and integrate across the two halves would perform well in congruent trials, but at chance levels in incongruent trials, which would result in a maximal CE. On the other hand, if the observer is able to maintain a part-based focus on only the target halves, performance would become equal in congruent and incongruent trials, thus, the CE would vanish.

Favoring a perceptual account of facial feature integration, one may be seduced to analyze only the “same” trials, and to disregard the “different” trials (Rossion, 2013, p. 42). However, ignoring performance achieved with one trial class may seriously confound perceptual and non-perceptual sources of the observer's decisions. In this context, it is important to note that the CD is only an experimental design and it does not favor any theoretical account of object processing. As outlined below, it is possible to derive testable hypotheses for the perceptual account of the composite effect within the CD. Advantageously, these hypotheses can be tested using bias-free measurements of performance in a same/different forced choice task.

### 1.2. Testing the perceptual and the decisional account of the composite effect with the complete design

Some authors regard the composite effect as a visual illusion that stems from perceptual integration of upper and lower faces halves (Rossion, 2008, 2013). To make the perceptual account of the composite effect more explicit, one may conceive an ideal “holistic” observer who refers to a whole face as the smallest perceptual unit when exposed to natural and intact face stimuli. However, this notion is just an ideal, because human observers can take a part-based focus of facial stimuli (Meinhardt-Injac et al., 2010, 2011). As outlined above, this observer would yield a large congruency effect in the complete design. Moreover, she/he would show a unique response pattern in incongruent trials (see Figure 1A). When exposed to the “same” trials, she/he should tend to respond “different” because the wholes formed by fusing the upper and lower haves are different. In the “different” trials she/he should also tend to respond “different” because the wholes are also different. That is, an observer who relies on the perceptual integration of the upper and lower halves should exhibit a strong response bias toward the “different” response category in incongruent trials. Conversely, in congruent trials, she/he should exhibit no response bias because the wholes are same in the “same” trials and different in the “different” trials. This means that a unique and testable prediction exists for the perceptual account of the congruency effect in the CD.

**Prediction 1**. *Suppose in a same/different face matching experiment in the complete design upper and lower face halves are perceptually integrated into a unified whole facial percept, and the observer relies on this percept in most of the trials when she/he decides about the identity of face halves. In this case a large congruency effect will exist with a strong bias toward “different” responses in incongruent trials and no bias toward either response category in congruent trials*.

This prediction has an important implication for the conclusions that can be drawn from the absence of response bias in incongruent trials. As it is implied by Prediction 1, a bias toward “different” responses in incongruent trials is a *necessary condition* for the perceptual account. If the bias is not observed, it can be concluded that the subject's response behavior is not guided by a unified whole facial percept (i.e., she/he is no “holistic” observer). On the other hand, when the scheme of results is observed as postulated by Prediction 1, it does not offer conclusive evidence that a unified whole facial percept underlies the response behavior because alternative sources may yield the same result. However, it is good evidence because the crucial observation is a complex one that comprises three coincident components.

Let us now turn to the alternative view that face halves are perceived and encoded as independent parts, but interact at the decisional level (Richler et al., 2008a,b). As far as we see, the kind of interaction at the decisional level has not yet been explicated such that testable predictions can be derived concerning the nature of response bias (see Discussion, in Cheung et al., 2008). This lack of explanation is clearly a drawback. However, as the researchers pointed out, the interaction of face halves is stronger for faces than for other objects and it occurs automatically, while non-face objects need training or aiding context (Gauthier et al., 2003; Richler et al., 2009a). The degree of part interaction is expected to increase with increasing object expertise (Gauthier and Tarr, 2002; Gauthier et al., 2003; Richler and Gauthier, 2013). From this, it follows that there should be a strong congruency effect for faces, but not for non-familiar non-face objects. For the nature of the bias, no specific prediction is possible with the explication of this theory.

As outlined above, the nature of errors in incongruent trials is particularly important to understand the way face halves interact. The “holistic” observer is not expected to be prone to wrongly saying “same” when the target halves are different because then the target halves *and* the wholes are different. Instead, she/he is prone to wrongly saying “different” when the target halves are same because the wholes are different. Hence, a case in which errors of both kind are equally likely in incongruent trials (i.e., there is no bias toward either response category) would offer strong evidence that the observer does not rely on an unparsed whole facial representation. However, a strong congruency effect means that the observer makes many errors in the incongruent trials. While the absence of a “different” bias in incongruent trials would speak against a perceptual account of holistic processing, comparisons with the results for non-face objects are necessary to decide whether the congruency effect might reflect, at least partly, response interference, as with the Stroop effect (Richler et al., 2009b). The involvement of a response interference should concern faces and non-face objects as well. However, if congruency effects were negligible for non-face objects but substantial for faces, this finding would speak against response interference and would suggest a decisional account for the interaction among the face halves (see Discussion).

### 1.3. Task constraints

The same/different matching task used to study the composite effect involves categorization at the individual level, which is an important task constraint (see Discussion). Schyns and colleagues (Smith et al., 2004; van Rijsbergen and Schyns, 2009) recorded the early perceptual and face selective N170 and the P300, which reflects activation involved in categorial decisions (Goodale and Milner, 1992), while subjects made categorial decisions about faces (e.g., gender, facial expression). They found evidence for face specific encoding at early stages, but not much selectivity for the diagnostic features of the given categorization task. Modulation by mostly task-relevant diagnostic features was found only for the P300. Measuring the selectivity for spatial frequencies showed that the N170 was sensitive to both low and high spatial frequencies, while the P300 responded mainly to the high spatial frequencies of task-relevant diagnostic features (Smith et al., 2004). From these results the authors concluded that categorial decisions about objects are made at a later stage that transforms and reorganizes detailed diagnostic features.

The findings of Schyns and colleagues indicate that variation of task constraints can offer valuable clues about the functional role and the locus of feature integration in face perception. The difficulty of ignoring irrelevant context can be modulated by informing the observer early or late in the trial which object parts are to be compared. With an early cue the observer can try to attend to only diagnostic features, and to ignore irrelevant context. When the cue comes late in the trial, the observer must encode relevant and irrelevant features, and recall only the relevant features at decision. Therefore, contextual influence should be larger in the late cue condition. Second, feedback about correctness can help the observer to control contextual influence, and to optimize attentional selection. In a recent study (Meinhardt-Injac et al., 2011) it was shown that observers were able to use trial-by-trial feedback to regulate the influence of irrelevant external context features on relevant internal features. Strong improvement in accuracy was observed compared to the no-feedback condition, indicating that feedback indeed helped observers to attend to the diagnostic features.

Because faces and non-face objects differ in their degree of part integration, early/late cueing and feedback should modulate the congruency effect differently for both stimulus categories. Contextual influence is expected to be moderate for non-face objects. Therefore, also the modulating influence of early/late cueing and feedback should be small. In contrast, congruency effects for faces are expected to be substantial. The temporal cue position and feedback should therefore be crucial for controlling the influence of irrelevant facial features.

Using the CD enables us to characterize the nature of feature integration by judging congruency effects along with response bias. In particular, the perceptual account of the composite effect can be tested within the framework of the CD. Additionally, variation of constraints for attending diagnostic features and providing feedback or not can be used as a further means to strengthen a differential results pattern for faces and non-face objects. In this study we demonstrate that the CD is suitable for revealing different processing schemes for face and non-face objects reliably.

## 2. Materials and methods

### 2.1. Study outline

As in a previous study using the CD (Richler et al., 2009c), we used a same/different face matching task in which a composite study image was shown for a longer time interval (800 ms), followed by a composite test image shown for a shorter time interval (433 ms, see **Figure 4**). A cue informed the observer which halves, the upper or lower, were to be attended. The observers' task was to decide, as accurately as possible, whether the cued halves were the same or different. One group of participants received acoustical trial-by-trial feedback about correctness, the other received no feedback. The temporal position of the target cue was varied to modulate the constraints for attending diagnostic features. When the target cue coincides with the study image, the observer can adjust his/her attentional focus to only the target half and maintain it throughout the trial. When the target cue comes briefly before the test, the observer must encode the whole stimulus at study and then shift his/her attention toward the target half at test. Hence, an effective part-based strategy is possible if the observer is certain from the beginning of the trial about which halves are to be matched (Riesenhuber et al., 2004; Riesenhuber and Wolff, 2009). With variations in feedback and temporal cue position it is possible to measure performance under conditions where observers have good attentional control and learning opportunities (cue at the beginning of the trial and trial-by-trial feedback) and measure that point at which attentional control is hampered and the decision behavior cannot be optimized by cognitive markers (no feedback and cue briefly before test image). These conditions should illuminate whether faces and non-face objects differ concerning the efficient extraction of diagnostic cues for identity matching of halves. If feature integration across halves is mandatory for faces, faces should be less efficient in this respect because the influence of irrelevant features remains, and interferes with piecemeal analysis.

### 2.2. Participants

Fifty one subjects participated in the experiment with face stimuli; 24 in the no-feedback group and 27 in the feedback group. 38 subjects participated in the experiment with non-face stimuli; 19 in the feedback and 19 in the no-feedback group. In all groups, the proportion of female participants was about 65%. All participants were undergraduate students of psychology at the Johannes Gutenberg University Mainz, ages spanned between 20 and 24 years. Subject had normal or corrected to normal vision, using corrective lenses in the latter case. All subjects were naive with respect to the purpose of the experiment. They were given course credit points for participation. The study was conducted in accordance with the Declaration of Helsinki. In detail, subjects participated voluntarily and gave written informed consent for their participation. In addition, participants were informed that they were free to stop the experiment at any time without negative consequences, and that their data would be removed from the panel. The data were analyzed anonymously.

### 2.3. Apparatus

The experiment was executed with Inquisit runtime units. Stimuli were displayed on NEC Spectra View 2040 TFT displays in 1280 × 1024 resolution at a refresh rate of 60 Hz. Screen mean luminance *L*_0_ was 100 cd/m^2^ at a michelson contrast of (*L*_*max*_ − *L*_*min*_)/(*L*_*max*_ + *L*_*min*_) = 0.98. No gamma correction was used. The room was darkened so that the ambient illumination matched that of the screen. Stimuli were viewed binocularly at a distance of 70 cm. Subjects used a distance marker but no chin rest throughout the experiment. Stimulus size was 250 × 350 pixels (width × height), which corresponded to 10 × 12.5 cm of the screen, or 8° × 10° measured in degree of visual angle at 70 cm viewing distance. Stimulus position jittered randomly within a region of ± 50 pixels around the center of the screen to preclude pixel matching strategies between two subsequent stimulus presentations. Masks subtended 350 × 450 pixels (width × height), and their position was always fixed at the screen center. They were constructed from randomly ordered 5 × 5 pixel blocks of the prior image shown. Subjects provided responses on an external key-pad, and wore light headphones for acoustical feedback in the feedback condition.

### 2.4. Stimuli

#### 2.4.1. Face stimuli

Photographs of 20 male models were used for stimulus construction. The models gave written consent for scientific use and publication of their face images. These photographs were frontal view shots of the whole face, captured in a professional photo studio under controlled lighting conditions. The original images were edited using Adobe Photoshop CS4 to generate the set of stimuli used in the experiment. Photographs were initially converted to 8 bit grayscale pictures and superimposed with an elliptical frame mask to obliterate all external facial features, such as hair, ears, or chin line. The elliptical cutouts were then split horizontally at the bridge of the nose, thus yielding 20 upper and 20 lower face halves. Each upper half was recombined with three lower halves to constitute a final set of 60 compound faces. The cutline between the face halves was concealed with a white bar 5 pixels in thickness. It was warranted that any upper face part was never recombined with the lower half of the same original face. In addition, each of the 20 lower and upper halves appeared exactly three times in the final set of stimuli.

#### 2.4.2. Non-face stimuli

**Figure 3:**
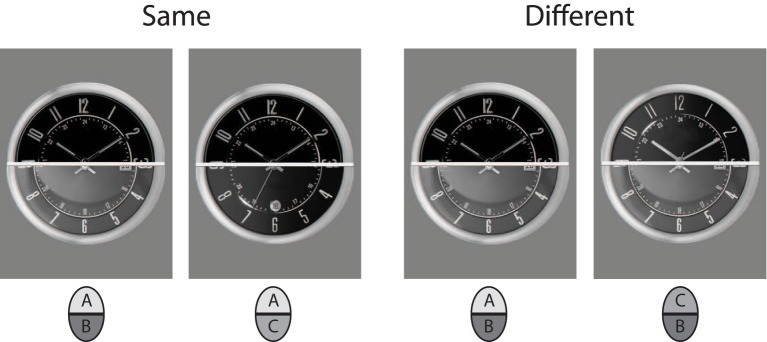
**Example of stimulus pairings in an incongruent trial with watch stimuli**.

Twenty watches were used for the non-face stimuli. Watches were sampled from internet sources, and selected such that they had high overall resemblance, showed the same time, and had non-salient distinctive single features. The images were transformed to gray and matched on lightness and contrast. The cutline for subdividing into upper and lower halves was exactly through the midpoint of the clock face. All external features were removed using a circular frame mask that contained only the clock face of the watches with numbers and hour hands. Stimulus examples are shown in Figure 3. As for the faces, a final set of 60 composite faces was constructed.

### 2.5. Procedure

**Figure 4:**
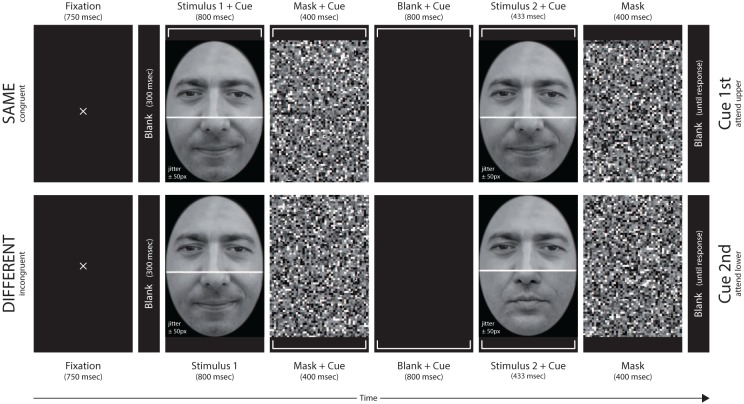
**Examples of a single trial for the cue 1st (upper row) and the cue 2nd (lower row) condition**. The upper row shows a same trial in congruent condition with upper target half, the lower row a different trial in incongruent condition with lower target half.

A same/different forced choice matching task was used. Subject were informed that face pairs could differ in the cued and non-cued halves and that object matching was to be done upon just the cued halves. The temporal order of events in a trial sequence was: fixation mark (750 ms)—blank (300 ms—study face stimulus (800 ms)—mask (400 ms—blank (800 ms—test face stimulus (433 ms)—mask (400 ms)—blank frame until response (see Figure 4). The allocation of participants to the feedback and the no-feedback group was random. Subjects were made familiar with the task by going through randomly selected probe trials to ensure that the instructions were understood and could be put into practice. All subjects completed two cue conditions. In the “cue 1st” condition a rectangular bracket marking the target face half was shown simultaneously with the study face, and remained until the test face was masked. In the “cue 2nd” condition the cue presentation began with the mask of the study face. A trial was deemed congruent (CC) when the non-cued half of the face was different in “different” trials and same in “same” trials, and it was considered incongruent (IC) when the non-cued half was same in “different” trials and different in “same” trials.

For each stimulus class the experimental design was a 2 (Feedback) × 2 (Cue position) × 2 (Congruency) × 2 (Target half) factorial plan. Feedback was implemented as a between-subjects factor; all others were within-subjects factors. Each condition was measured with 16 “same” and 16 “different” trials. Trials were shuffled and assembled in a randomly ordered measurement list, but with cue position ordered in blocks^1^. The two blocks, interleaved by a brief pause, were administered on a single day. Each block lasted about 15 min.

### 2.6. Dependent measures and data transformations

For the same/different experiment the “same” response category was defined as the target category. Accordingly, hit-rate (Hit) was defined as the rate of correctly identifying same target halves and correct rejection rate (CR) was defined as the rate of correctly identifying different target halves. False alarm rate (FA) and the rate of misses (Miss) were defined as being the complementary rates to CR and Hit, respectively. Rates were estimated by pooling across the relative frequencies obtained for upper and lower half matching. The relative frequency data were transformed into *d*′ according to

(1) $$d^{\prime }=z(CR)-z(Miss).$$

**Figure A1:**
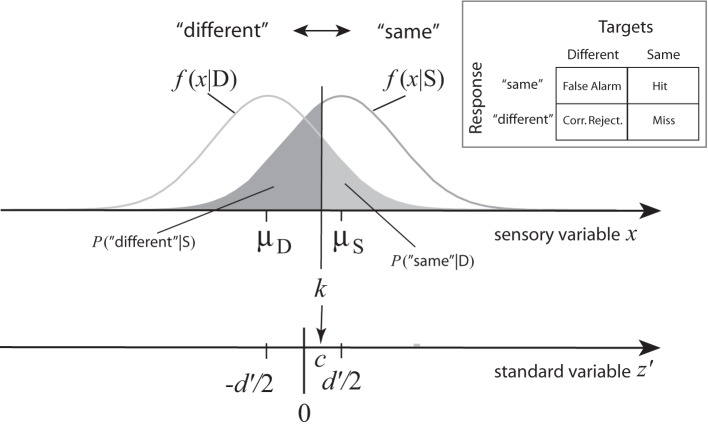
**Likelihood functions *f*(*x|D*), *f*(*x|S*) as normal probability density functions with equal variance σ^2^, decision criterion *k*, and corresponding probabilities of “false alarm” (*P*(“*same*”|*D*)) and “miss” (*P*(“*different*”|*S*)) events resulting from the position of the decision criterion *k* on the latent sensory continuum *x***. The lower continuum represents a transformed standard axis with *d*′/2 as the new origin. Expressed on this axis, positive values of the transformed decision criterion, *c*, correspond to more frequent “different” than “same” judgments, a *bias* toward the “different” response category (see arrow).

In Equation (1) *z* is the quantile of the standard normal distribution. If the standard scale is shifted leftward about *d*′/2, the fair response criterion is located at the origin (see Appendix). By calculating the response criterion *c* on this scale

(2) $$c=z(CR)-\frac{d^{\prime }}{2}$$

response bias can be evaluated because positive values of *c* mean that the observer prefers “different” responses, while negative values of *c* indicate that she/he prefers the “same” response category (see Figure A1).

A bias measure can alternatively be defined in terms of the error proportion:

(3) $$q=\frac{Miss}{Miss+FA}.$$

If *q* = 0.5, then both kinds of errors are made with the same frequency. A ratio of *q* > 0.5 indicates a tendency to say “different” while *q* < 0.5 indicates a preference toward “same” responses. The error proportion measure, *q*, has the advantage that it easy to interpret. For example, a value of *q* = 0.7 means that 70% of all errors are wrong “different” responses and 30% are wrong “same” responses.

A further way to assess response bias is to look at the odds-ratio statistics. The odds-ratio of both errors is defined

(4) $$OR=\frac{Miss/Hit}{FA/CR}.$$

The odds-ratio is a straightforward way to assess how much higher the odds are for wrong “different” responses compared to wrong “same” responses.

### 2.7. Data analysis

Agglomerating the rates for upper half and lower half matching resulted in *N* = 32 replications for each trial type. If CR or Miss rates were zero or unity, they were corrected to 1/(2*N*) and 1 − 1/(2*N*), respectively, before *d*′ data were calculated (MacMillan and Creelman, 2005, p. 8). The *d*′ data were analyzed with ANOVA with feedback as the grouping factor and cue position and congruency as repeated measurement factors. Separate analyses were carried out for faces and watches. Congruency effects were calculated from the *d*′ data by taking the difference *CE* = *d*′(*CC*) − *d*′(*IC*) on the level of individual subjects.

## 3. Results

### 3.1. Matching accuracy

**Figure 5:**
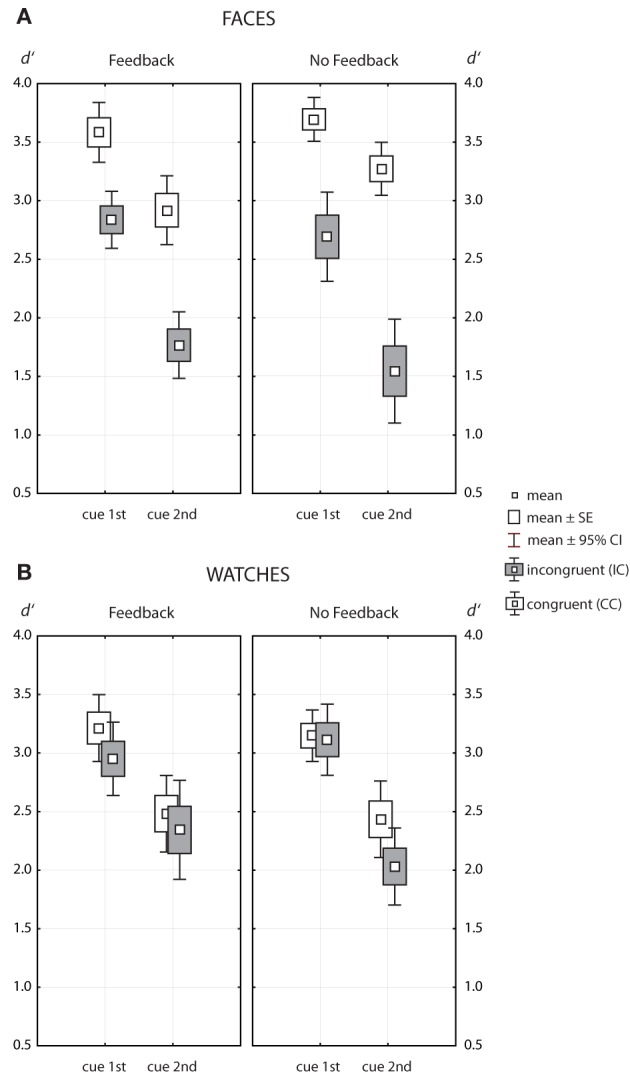
**Box-Whisker plots of the same/different matching accuracy measured in *d*′, for faces (A) and watches (B)**. Data for congruent contexts are indicated by open symbols, symbols filled with gray indicate data for incongruent contexts.

Figure 5 shows the data for faces and watches as Box-Whisker plots. Widely different results were obtained for faces and watches. The ANOVA results for faces (see Table 1) indicated a strong effect of cue position [*F*_(1, 49)_ = 88.8, *p* = 1.4 · 10^−12^, η^2^_*p*_ = 0.644] and a strong effect for congruency [*F*_(1, 49)_ = 132, *p* = 1.4 · 10^−15^, η^2^_*p*_ = 0.73]. The effect of congruency was strongly modulated by cue position [*F*_(1, 49)_ = 30.0, *p* = 1.5 · 10^−6^, η^2^_*p*_ = 0.379], and, to smaller degrees, by feedback [*F*_(1, 49)_ = 4.29, *p* = 0.044, η^2^_*p*_ = 0.081]. There was no main effect of feedback [*F*_(1, 49)_ = 0.03, *p* = 0.968, η^2^_*p*_ = 0.001], and cue position and feedback did not interact [*F*_(1, 49)_ = 0.22, *p* = 0.64, η^2^_*p*_ = 0.004].

**Table 1:** **ANOVA results for the same/different matching accuracy for faces (*d*′ measure)**.

**Source of variation**	***SS***	***df***	**$\hat{\sigma }$^2^**	***F***	***p***	**$\hat{\eta }$^2^**
Feedback (A)	0.03	1	0.03	0.03	0.868	0.001
Error	52.84	49	1.08			
Cue position (B)	34.71	1	34.71	88.76	0.000	0.644
Feedback × Cue position	0.09	1	0.09	0.22	0.640	0.004
Error	19.16	49	0.39			
Congruency (C)	68.06	1	68.06	132.76	0.000	0.730
Feedback × Congruency	2.20	1	2.20	4.29	0.044	0.081
Error	25.12	49	0.51			
Cue position × Congruency	4.06	1	4.06	29.96	0.000	0.379
*A* × *B* × *C*	0.32	1	0.32	2.39	0.128	0.047
Error	6.64	49	0.14			

*The table shows source of variation, sum of squares (*SS*), degrees of freedom (*df*), variance estimate ($\hat{\sigma }$^2^), *F*- ratio, (*F*), significance level, *p*, and partial eta-squared, η^*2*^_*p*_*.

For watches (see Table 2), there was a strong effect of cue position [*F*_(1, 36)_ = 107, *p* = 2.5 · 10^−12^, η^2^_*p*_ = 0.748] and a smaller effect of congruency [*F*_(1, 36)_ = 8.62, *p* = 0.006, η^2^_*p*_ = 0.193]. The latter effect did neither depend on cue position [*F*_(1, 36)_ = 0.57, *p* = 0.456, η^2^_*p*_ = 0.016], nor on feedback [*F*_(1, 36)_ = 0.02, *p* = 897, η^2^_*p*_ < 0.001]. As for faces, there was no main effect of feedback [*F*_(1, 36)_ = 0.15, *p* = 0.701, η^2^_*p*_ = 0.004], and feedback and cue position did not interact [*F*_(1, 36)_ = 2.30, *p* = 0.139, η^2^_*p*_ = 0.06].

**Table 2:** **ANOVA results for thesame/different matching accuracy for watches (*d*′ measure)**.

**Source of variation**	***SS***	***df***	**$\hat{\sigma }$^2^**	***F***	***p***	**$\hat{\eta }$^2^**
Feedback (A)	0.17	1	0.17	0.15	0.701	0.004
Error	39.70	36	1.10			
Cue position (B)	23.32	1	23.32	106.96	0.000	0.748
Feedback × Cue position	0.50	1	0.50	2.30	0.139	0.060
Error	7.85	36	0.22			
Congruency (C)	1.67	1	1.67	8.62	0.006	0.193
Feedback × Congruency	0.00	1	0.00	0.02	0.897	0.000
Error	6.98	36	0.19			
Cue position × Congruency	0.14	1	0.14	0.57	0.456	0.016
*A* × *B* × *C*	0.58	1	0.58	2.30	0.138	0.060
Error	9.06	36	0.25			

### 3.2. Congruency effects

**Figure 6:**
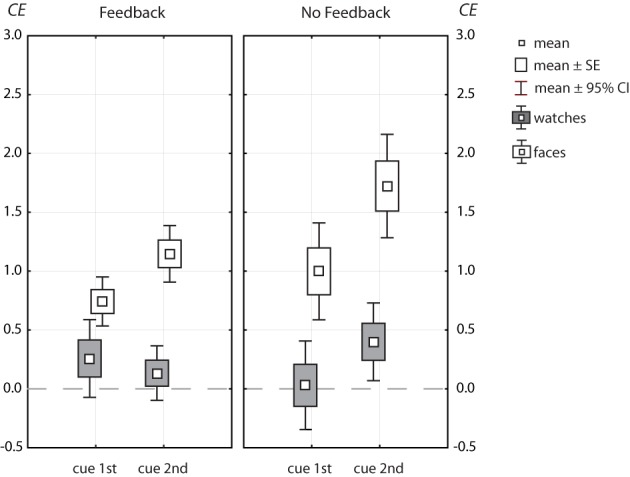
**Box-Whisker plots of congruency effects, *CE* = *d*′(*CC*) − *d*′(*IC*), for faces (open symbols) and watches (symbols filled with gray)**.

Figure 6 shows the congruency effects (CE) for faces (open symbols) and watches (filled symbols), as Box-Whisker plots^2^. A significant congruency effect in one condition, when the cue came at the second position in the absence of feedback, existed for watches [*CE* = 0.404, *t*_(18)_ = 2.969, *p* = 0.008]. The lack of any interactions of congruency with cue position or feedback (see above) indicates that these factors did not modulate the congruency effect (see Table 2). Further, the analysis yielded no interaction of all three factors [feedback × cue position × congruency, *F*_(1, 36)_ = 2.30, *p* = 0.138, η^2^_*p*_ = 0.06].

Congruency effects for faces were strong, ranging from about 0.75 *d*′ units (cue1st with feedback) to 1.75 *d*′ units (cue2nd without feedback). Congruency effects for faces depended largely on cue position, and were much larger when the cue came at the second position [Δ*CE* = 0.565, *F*_(1, 49)_ = 30.0, *p* = 1.5 · 10^−6^]. Congruency effects were also stronger without than with feedback [Δ*CE* = 0.416, *F*_(1, 49)_ = 4.29, *p* = 0.044]. Feedback and cue position were found to modulate the congruency effect independently, since the higher level interaction among all three factors failed to reach significance [feedback × cue position × congruency, *F*_(1, 49)_ = 2.39, *p* = 0.128, η^2^_*p*_ = 0.047].

Hence, we found a clear pattern for congruency effects. For watches, the data yielded a consistent tendency to perform better in congruent contexts compared to incongruent contexts (see Figure 5). However, congruency effects remained marginal clearly below half a *d*′ unit and did not depend on cue position or feedback. For faces, however, there were large congruency effects, which were strongly modulated by cue position (η^2^_*p*_ = 0.379), and, to minor degrees, by feedback (η^2^_*p*_ = 0.081).

### 3.3. Response bias

**Figure 7:**
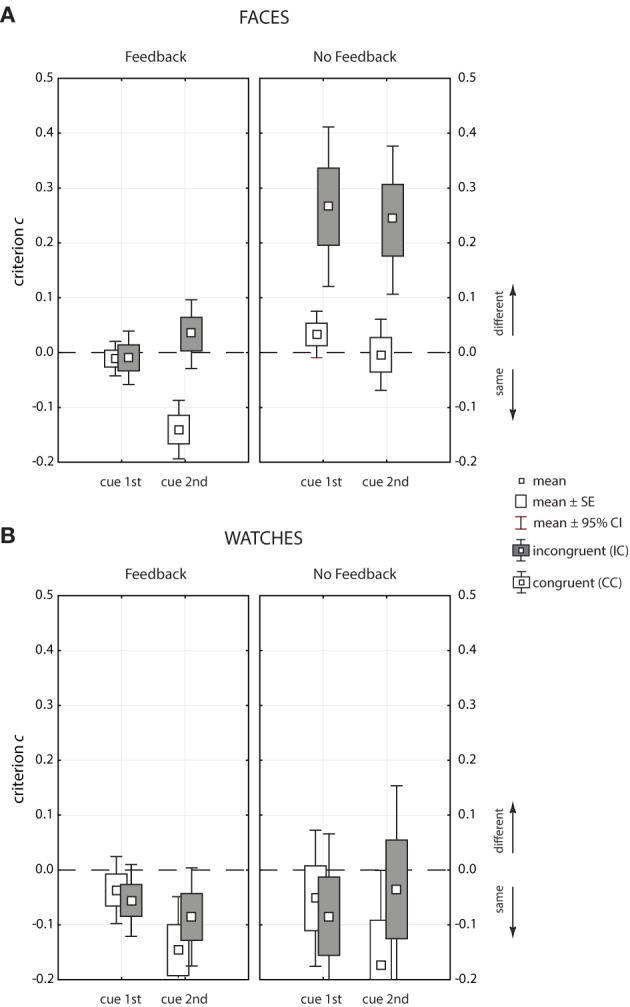
**Box-Whisker plots of the decision criterion *c* used to assess response bias for faces (A) and watches (B)**.

Figure 7 shows the response criterion *c* for faces (upper panels, A) and watches (lower panels, B) as Box-Whisker plots. Tables 3, 4 show detailed results, including both the *c* and the *q* measure, miss and false alarm rates, overall error rate *p*_*e*_, and odds ratio of misses and false alarms. To judge response bias statistically it has to be verified whether the mean *c* value is significantly above (“different” bias), or below (“same” bias) the expected value 0, as indicated by the Whiskers^3^. For faces, there was only one significant bias in the feedback condition (see left upper panel of Figure 7), where a tendency toward “same” responses existed for congruent trials when the cue came at the second position [*c* = −0.14, *t*_(26)_ = −5.03, *p* = 3.1 · 10^−5^]. There was no response bias in the absence of feedback in congruent contexts; however, a pronounced tendency toward “different” responses existed in incongruent trials [cue1st: *c* = 0.27, *t*_(23)_ = 5.19, *p* = 2.9 · 10^−5^; cue2nd: *c* = 0.24, *t*_(23)_ = 4.80, *p* = 7.6 · 10^−5^]. To judge bias it is also important how many errors occurred in a given condition because response bias is of practical relevance only if a substantial number of errors are made. This was the case for incongruent trials in the absence of feedback. Here, 14% misses stood against 5.3% false alarms when the cue came first (*p*_*e*_ = 9.7%), and 29.8% misses compared to 15.5% false alarms when the cue came at the second position (*p*_*e*_ = 22.6%). Response bias did not occur in the feedback condition in incongruent trials when the cue came at the second position, although the error rates were rather high (*p*_*e*_ = 18.8%, see last line of Table 3). Instead, there was “same” bias in congruent trials, but there, the error rate was moderate, with 9.3% false alarms compared to 5.5% misses (*p*_*e*_ = 7.4%, see 2nd last line of Table 3). This indicates that trial-by-trial feedback influenced the subjects' response strategies. Comparing the likelihood of both kind of errors with the odds-ratio statistics confirmed this result. In the absence of feedback and in incongruent trials the chance for wrong “different” responses was more than double the chance for wrong “same” responses when the cue came at the second position, and nearly threefold when the cue came at the first position. With feedback and in congruent trials the chance for wrong “different” responses was nearly halved when the cue came at the second position. All other odds-ratios are about 1, which indicates balanced chances for errors of both kinds.

**Table 3:** **Bias measure results for faces**.

**Feedback**	**Cue position**	**Congruency**	***c***	***s_*e*_***	***t***	***p***	**Miss (%)**	***FA* (%)**	***p_*e*_*(%)**	***q* (%)**	***OR***	***N***
N	1st	CC	0.03	0.018	1.87	0.075	3.5	3.0	3.2	53.8	1.17	24
N	1st	IC	0.27	0.052	5.19	0.000	14.0	5.3	9.7	72.5	2.90	24
N	2nd	CC	−0.01	0.029	−0.09	0.927	5.0	5.1	5.1	49.7	0.99	24
N	2nd	IC	0.24	0.051	4.80	0.000	29.8	15.5	22.6	65.8	2.32	24
Y	1st	CC	−0.01	0.017	−0.56	0.579	3.6	3.7	3.6	48.9	0.96	27
Y	1st	IC	−0.01	0.049	−0.17	0.865	7.7	7.9	7.8	49.2	0.97	27
Y	2nd	CC	−0.14	0.028	−5.03	0.000	5.5	9.3	7.4	37.0	0.56	27
Y	2nd	IC	0.04	0.048	0.73	0.472	19.8	17.9	18.8	52.5	1.13	27

*The table shows mean c value, its standard error, s_e_, t- value and probability that the expected value 0 is within the distribution of the mean c value, misses, false alarms, error rate, p_e_ = (Miss+FA)/2, and error proportion measure, q, on a percent scale, odds-ratio, OR, and sample size, N*.

**Table 4:** **Bias measure results for watches, See Table 3**.

**Feedback**	**Cue position**	**Congruency**	***c***	***s_*e*_***	***t***	***p***	**Miss (%)**	***FA* (%)**	***p_*e*_* (%)**	***q* (%)**	***OR***	***N***
N	1st	CC	−0.05	0.047	−1.10	0.287	5.2	6.4	5.8	44.9	0.80	19
N	1st	IC	−0.08	0.053	−1.57	0.133	5.0	7.0	6.0	41.7	0.70	19
N	2nd	CC	−0.17	0.067	−2.59	0.018	8.2	14.8	11.5	35.6	0.51	19
N	2nd	IC	−0.04	0.070	−0.50	0.624	14.6	16.3	15.5	47.3	0.88	19
Y	1st	CC	−0.04	0.047	−0.78	0.446	5.0	5.8	5.4	46.3	0.86	19
Y	1st	IC	−0.06	0.053	−1.03	0.315	6.3	7.7	7.0	44.7	0.80	19
Y	2nd	CC	−0.16	0.067	−2.39	0.028	8.0	13.9	11.0	36.5	0.54	19
Y	2nd	IC	−0.09	0.070	−1.21	0.242	10.4	13.8	12.1	43.0	0.72	19

For watches the *c* values were negative in all conditions, which indicates a global bias toward “same” responses. However, statistical significance was reached only in two conditions, congruent trials when the cue came at the second position, in the presence of feedback [*c* = −0.16, *t*_(28)_ = −2.39, *p* = 0.028], and in its absence [*c* = −0.17, *t*_(28)_ = −2.59, *p* = 0.018]. In both conditions a significant proportion of errors occurred (see Table 4). Testing first against second cue position for congruent trials revealed a stronger “same” bias at the second cue position in the presence of feedback [Δ*c* = 0.124, *F*_(1, 36)_ = 6.46, *p* = 0.015] and in its absence [Δ*c* = 0.123, *F*_(1, 36)_ = 6.31, *p* = 0.017]. In incongruent trials no corresponding differences were found [feedback: Δ*c* = 0.03, *F*_(1, 36)_ = 0.43, *p* = 0.518; no feedback: Δ*c* = −0.05, *F*_(1, 36)_ = 1.12, *p* = 0.296]. A global bias toward “same” responses also became apparent in the mean odds-ratio, which was 0.73, indicating that wrong “different” responses had about a three quarters chance to occur compared to wrong “same” responses. In the two conditions where a significant bias measure *c* was observed this chance fell to about 0.5. This shift from the general balance of chances observed for watches was by far not as strong as the three shifts of chances observed in the face matching experiment.

## 4. Discussion

Testing the effects of congruency, target certainty, and feedback in a same/different matching task showed strong effects of congruency and target certainty, while feedback yielded no effects on overall matching performance. This finding was the case for faces and watches. The magnitude of the congruency effects, however, differed widely between the two object classes. For watches, congruency effects were consistently present in all conditions, but marginal, and reached significance only in the condition where subjects could not prepare well for the task (no feedback and late target cue). For faces, in contrast, there were large congruency effects, which were substantial when subjects could prepare well for the task (feedback and target cue already at study) and very large if not (no feedback and late target cue). For faces, feedback and target certainty modulated the congruency effect independently (additively), while no modulatory influence of these factors was found for watches. Hence, the magnitude of congruency effects and the pattern of their dependency on feedback and target half certainty clearly separated facial from the non-facial watch stimuli.

Analysis of response bias also revealed differential result patterns for faces and watches. For faces, response bias strongly depended on the feedback condition, while, for watches, feedback did not influence the nature of response preferences. For faces, a strong “different” bias was observed in the absence of feedback in incongruent trials, and no response preference was found in congruent trials. With feedback, the “different” bias vanished completely, but a “same” bias emerged in congruent trials and when the cue came at the second position. For watches, a marginal, but general “same” bias was found, which was significant in congruent trials when the cue came at the second position. Hence, a “different” bias in incongruent trials, which might be diagnostic of a “holistic” representation, which interferes with proper comparison of parts, was only found for faces. Indeed, the finding of the large congruency effects, together with a strong “different” bias only in incongruent trials, came out in the no-feedback condition where no external signals communicated to the observer that she/he erroneously judged face halves as different. This finding is strong support for the perceptual account of the composite effect (see Introduction).

### 4.1. Target certainty

Subjects made more errors when they were informed about the target half briefly before test. However, the effect of cue position was differential for congruent and incongruent trials only for faces, not watches. For faces, the need to change the attentional focus within a trial impaired performance to larger degrees in incongruent trials, thus enlarging the congruency effect (see Figures 5, 6). As stated above (see Materials and Methods) the reasoning behind the manipulation of cue position was to probe whether the congruency effect depended on how the subjects prepared for the task. While it was a reasonable assumption that a priori knowledge and the opportunity to adopt a viewing strategy in advance would regulate the face processing mode (Riesenhuber et al., 2004; Riesenhuber and Wolff, 2009), our results for the bias measure only partly support this claim. Regardless of feedback, subjects could try to encode and compare only the target face half when the cue came before the trial. While subjects made more errors mostly in incongruent trials when the cue came late in the trial (see Table 3), the strong bias in favor of “different” judgments was the same for both cue positions when there was no feedback. Hence, the opportunity to adjust the attentional focus in advance clearly reduced the absolute number of errors in incongruent trials, but it did not change their nature. This finding suggests that the early cue enabled a part-based viewing strategy in more of the trials, but errors still came from global contextual influence.

### 4.2. Feedback

The results of this study showed that the effects of feedback are highly differential for faces and watches. For watches, providing feedback or not did not have much effect. Feedback did not modulate performance, it did not modulate the congruency effect, and it did not change the nature of response preferences in any respect. For faces, feedback did not modulate the general level of matching accuracy; however, it did modulate the congruency effect and it changed observers' response preferences qualitatively (see Figure 7). With feedback, the response bias pattern that suggested a perceptual account of the congruency effect in the no feedback condition was lost. This finding indicates that, with trial-by-trial feedback, observers adjusted either their perceptual or decisional strategies. In the following, we argue that subjects adjusted mostly their decisional strategies.

If observers frequently resort to the “different” response in incongruent trials, trial-by-trial feedback would signal her/him that she/he overlooked the sameness of the target halves, which should initiate a more careful use of the “different” button in the course of the experiment. We noted above that feedback reduced the matching errors particularly in incongruent trials, which limited the congruency effect (see Table 3). However, overall face-matching performance was the same with and without feedback. This can only happen if performance in congruent trials worsens in the presence of feedback, which was exactly the case. Table 5 shows the pairwise comparisons of performance with and without feedback. The results confirm that performance slightly worsened in congruent trials and slightly improved in incongruent trials; however, none of these changes reached statistical significance. Indeed, the largest change was worsening of performance in congruent trials when the cue came at the second position, which just failed to reach significance. These results show that the smaller congruency effects in the feedback condition (see Results) were artifacts of change in opposite directions for congruent and incongruent trials. In fact, no net improvement of performance occurred by providing trial-by-trial feedback.

**Table 5:** **Pairwise tests for face-half matching with and without feedback**.

**Cue position**	**Congruency**	***d*′ (*FB*)**	***d*′ (*NoFB*)**	**Δ*d*′**	***s_*e*_***	***t***	***p***
1st	CC	3.59	3.70	−0.11	0.157	− 0.71	0.483
1st	IC	2.84	2.69	0.15	0.214	0.68	0.502
2nd	CC	2.92	3.27	−0.35	0.184	−1.93	0.060
2nd	IC	1.77	1.55	0.22	0.250	0.89	0.378

When we look at the changes in the nature of errors, the conclusion that feedback led only to a change of decisional strategy is further substantiated. The net error rate *p*_*e*_ was mostly comparable in two corresponding conditions with and without feedback; however, there was an overall shift in bias toward more “same” responses with feedback. In the cue 1st condition, this did not occur because errors occurred only occasionally.

Change of response criteria, but lack of net performance improvement, indicates that feedback could not be used to refine a perceptual strategy with better attentional control of the unattended face halves. However, Meinhardt-Injac et al. (2011) reported this function of feedback in controlling the contextual effects of external features on internal target features. There, subjects were able to use feedback for improvement in incongruent trials, while performance in congruent trials remained as good as that in the without feedback condition. However, the task in Meinhardt-Inajc and colleagues' study was less complex and did not require attentional shift within a trial, which is a difficult task (Lincolt et al., 1997). In addition, learning to regulate the influence of incongruent context information was easier because it could be achieved by learning to better focus the inner face parts and ignore the facial surroundings.

### 4.3. Response bias for watch stimuli

The discussion in the foregoing section showed that the response bias results for faces can be explained by the perceptual account of the composite effect in cases with no external markers that might alert subjects to the fact that they falsely think the halves are different. When such external markers were provided, subjects changed their response strategies and relabeled perceptual states as “same,” which they formerly labeled “different”. Because the performance measure in the complete design was bias-free, the findings suggest that this strategy was decisional and did not lead to net change of performance.

In addition to feedback and reward, a further factor that might influence response bias is the stimulus material. Figure 7A shows that faces in the congruent trials were judged as “same” or “different” with practically equal likelihood when there was no feedback. This finding indicates that the stimulus material was well balanced in this respect. Composite watch stimuli, however, had to be constructed from exemplars with high overall similarity. These stimuli differed by single details, otherwise the matching task would have been too easy. The bias data (see Results, see Figure 7B and Table 4) show that subjects had a general tendency to overlook the crucial details, which made the difference. This finding was independent of the congruency relation; however, occurred more frequently when the cue came at the second position. This is plausible because, with an additional attentional shift, finding the crucial feature in 400 ms is more difficult. In the cue 1st condition, the search was restricted to just one half.

### 4.4. The congruency effect in the complete design

The findings of the present study support a perceptual account of the congruency effect for faces because congruency effects coincided with a response preference for “different” responses in incongruent trials, as is expected from the composite face “illusion” (Rossion and Boremanse, 2008; Rossion, 2013). These findings are consistent with recent findings of Gao et al. (2011) who used the CD to study the effect of priming local vs. global processing levels with Navon primes prior to composite face matching. Instead of using non-face controls, they compared congruency effects and response bias in aligned and misaligned arrangements of face halves. As in this study the authors found strong congruency effects which were accompanied by a “different” bias only in the incongruent trials for the aligned arrangement. For the misaligned arrangement, both the congruency effect and the bias vanished. Hence, currently there are two studies which used the CD and obtained results in agreement with the “holistic” encoding hypothesis for face stimuli, while non-face stimuli or misaligned faces yielded different result patterns in the combined effects of congruency and response bias.

Gauthier and colleagues also reported larger congruency effects for faces than for non-face objects (Gauthier et al., 2003; Richler et al., 2009a; Richler and Gauthier, 2013); however found mixed results for the nature of the bias. Cheung et al. (2008) reported a “different” bias for full-spectrum faces and low-pass filtered faces, and a “same” bias for high-pass filtered faces. In a series of experiments with arrangements similar to this study, also a “different” bias was observed for the late cue condition (Richler et al., 2008b, Exp. 1 and Exp. 3). However, in a later replication with different timings a “same” bias was reported (Richler et al., 2009c). From our estimation, a “same” bias is not easily explained in terms of facial feature integration. A preference for “same” responses in incongruent trials would mean that a subject more often indicates sameness of composite faces whereas both the target halves *and* the wholes formed by an integration of the halves differ. A possible explanation for a “same” bias could be that face part interaction enters in the calculation of an internal, multi-feature similarity measure. Since partly different (incongruent) is less than totally different (congruent), it could well be that the observer shows no “different” bias in incongruent trials when she/he is conservative with the response criterion on the latent similarity scale^4^. Because the authors currently decline from a unique interpretation of response bias (Cheung et al., 2008), a theoretical gap exists that should be closed by an explication of the rules for the interaction of independently encoded parts at the decision stage.

### 4.5. The use of task-relevant object information

At the individual level of categorization single facial features, their configural relationship (Leder and Bruce, 2000; Leder et al., 2001), and global face features such as skin texture and hue (Hancock et al., 2000; Meinhardt-Injac et al., 2013) can, in principle, be diagnostic for match or mismatch. This is a major difference to the categorization experiments of Schyns and colleagues (Smith et al., 2004; Joyce et al., 2006), where it was a priori clear that the inner face region around the eyes was most diagnostic for the gender discrimination task and the mouth region for the facial expression discrimination task. Certainly, face-halve matching with randomly changing target definitions between upper and lower halves also requires one to separate these two highly diagnostic face regions. However, our results are disappointing with respect to a better use and sharpening of diagnostic information with facilitative task demands. Results for the early cue show that selective encoding and comparing of diagnostic features was possible only for watches. For faces, the influence of the irrelevant face halves remained substantial, even though the observer could try to encode just one half. The results for the influence of feedback also show that faces and watches differ in the effective use of relevant cues. For faces, there was no learning because improvement in incongruent trials was achieved at the cost of impairment in the congruent trials. For watches, the small but significant congruency effect in the late cue condition vanished when feedback was provided; however, performance in congruent trials stayed the same. Absence of congruency effects and learning to improve in incongruent contexts showed that observers succeeded in retrieving diagnostic feature information mostly for watches.

Results obtained with the bubbles-technique (Gosselin and Schyns, 2001) suggest the presence of both diagnostic and less diagnostic features for faces at the early perceptual level, which indicates an automatic, task-independent mechanism for faces (Smith et al., 2004; van Rijsbergen and Schyns, 2009). The authors showed task-related modulation of the late P300 by demonstrating that the potential became more negative when the task-diagnostic features were faded in, and less negative when the task-diagnostic features of the concurrent task were present. This finding might indicate task-related feature selection at later stages. However, these two groups of facial features were presented individually. For the problem addressed here, it would be interesting to see how much negativity is reduced when the task irrelevant features are added. Comparing relative changes of both the N170 and P300 would indicate where feature integration among relevant and irrelevant features is stronger; at encoding or at decision. This is left to forthcoming experimentation.

## 5. Conclusions

It has been shown that the complete design can be used to derive testable predictions for the mechanisms of facial feature integration, which can be contrasted against results for non-facial objects. In studying the composite effect, the CD is highly recommended, since it ensures that the number of same and different face half pairings is fully balanced across attended and non-attended halves. Because of the high theoretical importance of the nature of response bias, use of a fully balanced design is mandatory, and it should be excluded that a response bias is induced merely by an unequal number of same and different face halves. With respect to the uniqueness of the congruency effect, the findings regarding the effects of feedback have revealed a weakness of the CE; therefore, we recommend not relying on only a difference measure (CE) when judging the effects of the congruency manipulation. Performance in incongruent trials is certainly more sensitive to task demands, but also sensitivity for congruent trials must be monitored, since there may be change in opposite directions. As an alternative that avoids some disadvantages of difference scores (Peter et al., 1993), regression-based techniques could be used (DeGutis et al., 2013). However, the initial empirical comparisons indicate no higher reliability of the regression method. Because bias-free performance measures are linked to the CD, it allows researchers to assess performance and response bias independently. As a formal framework for experimental design, the CD is neutral regarding divergent theoretical accounts of feature integration. Therefore, we consent to Richler and Gauthier (2013) in that the CD is, at the time, the right framework for studying the composite effect.

## Author contributions

All authors contributed equally to conception and design of the study. Malte Persike conducted the experiments and data preparation. Günter Meinhardt contributed data analysis and interpretation. All authors were involved in writing, preparation of the manuscript and its final approval. All authors agree to be accountable for all aspects of the work in ensuring that questions related to the accuracy or integrity of any part of the work are appropriately investigated and resolved.

### Conflict of interest statement

The authors declare that the research was conducted in the absence of any commercial or financial relationships that could be construed as a potential conflict of interest.

## Footnotes

^1^Pilot measurements showed that having cue position randomly interleaved rendered the task too difficult.

^2^Note that, since the CE is defined as a difference measure (see Materials and Methods), congruency effects are significant at the 5% alpha level if 0 is outside the confidence interval of the mean, which is easily seen from the Whiskers. We do not report ANOVA tables for the CE measure, since the results are identical with those for all interactions involving congruency at the original *d*′ data (see Tables 1, 2). We report results from pairwise tests necessary to judge differences in the magnitude of the CE. However, also these tests coincide with the tests for the interactions involving the congruency factor, since congruency and feedback have only 2 levels.

^3^Note that ANOVA of the *c* data does not indicate whether the values deviate significantly from 0. Therefore, results of separate *t*- statistics for each condition are listed in Tables 3, 4.

^4^We thank Peter Hancock for drawing our attention to this interpretation.

## Appendix: the bias measure *c*

Let us define the four events resulting from a 2 × 2 stimulus-response matrix with “same” and “different” as the response alternatives (see Figure A1) in terms of conditional probabilities:

$$\begin{array}{l} CR=P(“\text{different}”|D)\\ FA=P(“\text{same}”|D)\\ Miss=P(“\text{different}”|S)\\ Hit=P(“\text{same}”|S) \end{array}$$

According to the basic assumptions of signal detection theory, these probabilities derive from normal probability density function (likelihood functions), *f*(*x|D*) and *f*(*x|S*), with equal variance σ^2^. For the difference of the means of both distributions we have

$$\Delta \mu =\mu_{S}-\mu_{D}=k-\mu_{D}+\mu_{S}-k$$

where *k* is the decision criterion on the sensory continuum *x*, which is assumed to be constant throughout all measurements. Dividing by σ

$$\begin{array}{l} d^{\prime }=\frac{\Delta \mu }{\sigma }=\frac{k-\mu_{D}}{\sigma }+\frac{\mu_{S}-k}{\sigma }\\ \;=z_{D}-z_{S}\;=\Phi^{-1}\left(CR\right)-\Phi^{-1}\left(Miss\right). \end{array}$$

Here, Φ^−1^ is the inverse distribution function (quantile function) of the standard normal distribution, *z*_*D*_ is the standard quantile of *k* relative to *f*(*x|D*), and *z*_*S*_ is the standard quantile of *k* relative to *f*(*x|S*). Now, verify that standardization of *x* with respect to *f*(*x|D*) maps μ_*D*_ ↦ 0 and μ_*S*_ ↦ *d*′, i.e.,

$$z(\mu_{D})=\frac{\mu_{D}-\mu_{D}}{\sigma }=0\;z(\mu_{S})=\frac{\mu_{S}-\mu_{D}}{\sigma }=d^{\prime }.$$

The standardization *z* = (*x* − μ_*D*_)/σ may be shifted to a new origin, chosen as half the standardized distance of means, *d*′:

$$z^{\prime }=z-\frac{d^{\prime }}{2}.$$

This scale is chosen to express the response criterion *k* on a transformed standard axis:

$$c=z_{D}-\frac{d^{\prime }}{2}.$$

On this scale, positive values of *c* mean that the response criterion is closer to μ_*S*_, and negative values mean that it is closer to μ_*D*_. The means transform *z*′(μ_*D*_) = −*d*′/2, and *z*′(μ_*S*_) = *d*′/2, respectively.
